# Association of Impaired Cytochrome P450 2D6 Activity Genotype and Phenotype With Therapeutic Efficacy of Primaquine Treatment for Latent *Plasmodium vivax* Malaria

**DOI:** 10.1001/jamanetworkopen.2018.1449

**Published:** 2018-08-31

**Authors:** J. Kevin Baird, Melva Louisa, Rintis Noviyanti, Lenny Ekawati, Iqbal Elyazar, Decy Subekti, Krisin Chand, Anggi Gayatri, Instiaty, Saraswati Soebianto, Chelzie Crenna-Darusallam, Dwi Djoko, Bambang Dwi Hasto, Dubel Meriyenes, David Wesche, Erni J. Nelwan, Inge Sutanto, Herawati Sudoyo, Rianto Setiabudy

**Affiliations:** 1 Centre for Tropical Medicine and Global Health, Nuffield Department of Medicine, University of Oxford, Oxford, United Kingdom; 2 Eijkman-Oxford Clinical Research Unit, Eijkman Institute of Molecular Biology, Central Jakarta, Indonesia; 3 Department of Pharmacology, Faculty of Medicine Universitas Indonesia, Jalan Salemba Raya No. 6, Central Jakarta, Indonesia; 4 Eijkman Institute of Molecular Biology, Central Jakarta, Indonesia; 5 Army Health Command, East Jakarta, Indonesia; 6 Certara Strategic Consulting, Princeton, New Jersey; 7 Division of Tropical Infectious Diseases, Faculty of Medicine, Universitas Indonesia, Central Jakarta, Indonesia; 8 Department of Parasitology, Faculty of Medicine, Universitas Indonesia, Central Jakarta, Indonesia

## Abstract

**Importance:**

Latent hepatic *Plasmodium vivax* hypnozoites provoke repeated clinical attacks called relapses. Only primaquine phosphate kills hypnozoites, and its therapeutic activity may depend on naturally polymorphic cytochrome P450 2D6 isotype (CYP2D6) activity.

**Objective:**

To examine the association of impaired *CYP2D6* genotypes and CYP2D6 metabolic phenotypes with therapeutic failure of directly observed high-dose primaquine treatment for *P vivax* malaria relapse.

**Design, Setting, and Participants:**

Nested case-control study of patients who, in July 2014, completed a randomized clinical trial of directly observed primaquine treatment for radical cure of acute *P vivax* malaria in an area of Indonesia where reinfection during 1 year of posttreatment follow-up was improbable. A total of 177 of 180 patients with *P vivax* malaria completed the clinical trial of primaquine treatment to prevent relapse; 151 were eligible for recruitment as controls. After screening, 59 potential control individuals (no relapse) and 26 potential case patients (relapse) were considered, and 36 controls and 21 cases were enrolled.

**Exposures:**

Cases and controls were exposed to *P vivax* malaria and primaquine therapy but had variable exposure to the enzymatic activity of CYP2D6, classified as impaired by a genotype-determined qualitative phenotype (poor or intermediate), genotype-determined activity score less than 1.5, or a log of the 24-hour pooled urine dextromethorphan-dextrorphan metabolic ratio greater than −1.0.

**Main Outcomes and Measures:**

Unadjusted odds ratios (ORs) of relapse with impaired CYP2D6 metabolism determined by genotype or measured by urinary dextromethorphan-dextrorphan metabolic ratio.

**Results:**

Among the 21 cases (mean [SD] age, 30.5 [6.3] years; all male) and 36 controls (mean [SD] age, 29.0 [3.6] years; all male), 6 *CYP2D6* alleles (*1, *2, *4, *5, *10, and *41) occurred as 12 distinct genotypes, with model activity scores ranging from 0.0 to 2.0. Among 32 patients with genotypic activity scores of 1.0 or less, 18 had experienced relapse, whereas among the 25 with scores higher than 1.0, 3 had experienced relapse (OR, 9.4; 95% CI, 2.1-57.0; *P* = .001). When the log of the metabolic ratio of dextromethorphan-dextrorphan was −1.0 or less, only 1 of 18 patients experienced relapse, whereas above that threshold (consistent with low metabolic activity), 20 of 39 patients experienced relapse (OR, 18; 95% CI, 2.2-148.0; *P* = .007).

**Conclusions and Relevance:**

Genotype-determined and directly measured impaired levels of CYP2D6 activity were associated with elevated risk of therapeutic failure. These findings suggest a natural variability in CYP2D6-dependent metabolism of primaquine as a key determinant of therapeutic efficacy against latent *P vivax* malaria.

## Key Points

**Question:**

How is natural variation in cytochrome P450 2D6 activity associated with therapeutic efficacy of primaquine phosphate against latent *Plasmodium vivax* malaria?

**Findings:**

In this nested case-control study of 57 patients who had participated in a clinical trial of primaquine for radical cure of acute *P vivax* malaria, exposure to low levels of cytochrome P450 2D6 activity determined by genotype or measured by dextromethorphan metabolism phenotype was associated with a significantly increased likelihood of relapse of malaria in the year after directly observed high-dose primaquine therapy.

**Meaning:**

Impaired cytochrome P450 2D6 activity was significantly associated with high risk of therapeutic failure of primaquine, and this finding suggests cytochrome P450 2D6 involvement in producing a therapeutically active metabolite.

## Teaser

This nested case-control study explores the association of impaired CYP2D6 genotype and metabolic phenotype activity with failure of high-dose primaquine treatment for *Plasmodium vivax* malaria relapse in Indonesian men.

## Introduction

The human malaria parasite *Plasmodium vivax* may be exposed to 2.8 billion people and causes infection in millions annually.^1^ Historically considered intrinsically benign, *P vivax* has recently been reported to be associated with severe anemia, severe thrombocytopenia, respiratory distress, renal or hepatic dysfunction, seizures or coma, and shock.^2,3^ *Plasmodium vivax* malaria during early pregnancy has caused elevated risk of low birth weight, stillbirth, and spontaneous abortion.^4^ Prevention of such morbidity and mortality requires prompt diagnosis and effective chemotherapy.

This species, unlike the other major cause of human malaria, *Plasmodium falciparum*, activates dormant stages in the liver called hypnozoites derived from bradysporozoites inoculated by infectious anopheline mosquitoes. Each of the often multiple recurrent attacks caused by these hypnozoites in the weeks, months, and few years after infection create a risk of poor clinical outcomes and continued transmission. Among 2495 repatriated US soldiers infected by *P vivax* in the Pacific during World War II, the median number of attacks after removal from endemic areas was 10 to 14, with approximately equal minorities experiencing 1 to 3 or more than 20 attacks.^5^ A total of 90% to 96% of clinical attacks of *P vivax* in Thailand and 82% in Papua New Guinea, where *P vivax* infection is endemic, derived from an estimated 5 relapses per infectious event.^6,7^ Effective treatment of hypnozoites represents a therapeutic aim of great clinical and public health importance.^8^

Treatment of *P vivax* malaria requires distinct blood schizontocidal therapy against an acute attack and hypnozoitocidal therapy against latency.^9^ Chloroquine phosphate and primaquine phosphate have been those primary therapies since 1952. Primaquine is a problematic therapy, causing potentially fatal hemolytic anemia in patients with glucose-6-phosphate dehydrogenase (G6PD) deficiency—an inherited X-linked abnormality that occurs in approximately 8% of people living at risk of malaria.^10^ Resistance to chloroquine by *P vivax* commonly occurs, and artemisinin-combined blood schizontocides are effective therapeutic options^11^; however, primaquine remains the only hypnozoitocidal option.

Ascertaining therapeutic success vs failure of primaquine is fraught with uncertainty. Potentially important confounding factors must be considered: (1) many pharmaceutical companies of variable standards manufacture primaquine; (2) unsupervised adherence to the 14-day regimen is notoriously poor; (3) many countries recommend a less effective, lower total adult dose (210 vs 420 mg) in mitigating risk of harm in G6PD deficiency; (4) no technology distinguishes relapses from new primary attacks; and (5) risk and timing of relapse vary widely among geographic regions.^7^

The complex metabolism of primaquine includes generation of highly reactive quinonimine metabolites via cytochrome P450 2D6 (CYP2D6) enzymatic activity^12,13^ linked to therapeutic activity against hepatic schizonts of rodent plasmodia.^14^ CYP2D6 pharmacogenetics has been extensively investigated as metabolizing approximately 20% of prescribed medicines.^15,16,17^ The identity and source of the primaquine metabolite active against hypnozoites in humans remain unknown, but intermediate or poor metabolizer *CYP2D6* genotypes occurred with therapeutic failure in at least 3 patients not exposed to risk of reinfection during prolonged follow-up.^18,19^ Examining possible linkage of impaired CYP2D6 metabolism to primaquine treatment failure requires ruling out other likely causes. Patients given directly observed high-dose primaquine therapy and followed up for months free of reinfection are candidates for such studies.

In 2 clinical trials of primaquine in Indonesia, 252 patients received directly observed high-dose primaquine against latent *P vivax* infection, and 35 (13.9%) experienced relapse during a year of follow-up without risk of reinfection.^20,21^ In the current study, we evaluated CYP2D6 polymorphisms that occurred among patients of one of those trials.^21^ A nested case-control design used *CYP2D6* genotyping^22^ and measurement of CYP2D6-dependent metabolism of dextromethorphan to dextrorphan^23,24,25^ among 21 of 26 patients who experienced relapse (cases) compared with a random sample of 36 of the 151 patients who did not experience relapse (controls). We thus assessed the risk of relapse associated with exposure to genotype-determined or measured impaired levels of CYP2D6 metabolic activity. Implication of the enzyme responsible for the hypnozoitocidal metabolite of primaquine represents a key step in understanding the molecular basis of this important chemotherapy and the causes of its frequent clinical failure.

## Methods

### Setting

At an Indonesian army base at Sragen, Central Java, Indonesia, during July 2014, patients successfully completing a randomized clinical trial of primaquine phosphate therapy (0.5 mg/kg daily for 14 days) against relapse of *P vivax* malaria acquired in eastern Indonesia^21^ were screened for this study. They had been randomized to receive blood schizontocidal therapy for the acute attack with artesunate, dihydroartemisinin-piperaquine, or artesunate-pyronaridine and were predominantly ethnic Javanese Indonesian men. Indonesia excludes women from duties within deployed infantry battalions; therefore, no women were available for enrollment in these studies. The Ethics Committee for Health Research, Faculty of Medicine, Universitas Indonesia approved a protocol for this study on April 28, 2014. The Oxford Tropical Research Ethics Committee approved the protocol on May 8, 2014. The approved protocol describing this nested case-control study was registered on May 16, 2014.^26^ All patients provided written informed consent to participate in this study. The data were deidentified. This study accorded with the principles and provisions of the International Conference on Harmonisation Tripartite Guideline for Good Clinical Practice and the Declaration of Helsinki, whichever afforded greater protection of patients. Reporting of all aspects of this study adhered to the Strengthening the Reporting of Observational Studies in Epidemiology (STROBE) reporting guidelines for case-control studies.^27^

### Study Design and Population

A nested case-control design assessed the risk of therapeutic failure of directly supervised high-dose primaquine therapy against relapse of *P vivax* infection associated with exposure to low *CYP2D6* genotype–determined activity scores^22^ or CYP2D6-mediated metabolism of dextromethorphan.^23,24,25^ Cases experienced a relapse of *P vivax* malaria in the year after primaquine therapy during which reinfection did not occur. Unmatched, randomly selected controls were patients from the same trial who received the same treatments^21^ and did not experience relapse during 12 months of follow-up (eFigure 1 in the Supplement). Primaquine plasma concentrations from the clinical trial^21^ were measured and analyzed as detailed in the eAppendix in the Supplement.

### Inclusion and Exclusion Criteria

All screened patients were healthy male soldiers who resided at the study site who successfully completed participation in the clinical trial of primaquine treatment.^21^ They were fully recovered from the malaria attacks that had occurred 3 to 10 months previously and were available and willing to spend 48 hours in the research ward. Ineligible patients were unavailable or unwilling to participate or had abnormal clinical or laboratory findings during screening. Cases and controls were selected for recruitment on the basis of primaquine failure or apparent success, respectively. A total of 177 of 180 patients with *P vivax* malaria completed the clinical trial of primaquine against relapse; 151 were eligible for recruitment as controls. After screening, 59 potential controls (no relapse) and 26 potential cases (relapse) were considered. Among the 26 patients eligible for recruitment as cases, 2 were absent, 3 declined consent, and 21 volunteered. Among the 151 patients eligible as controls, a computer-randomized listing guided recruitment in listed order until 36 volunteered and successfully enrolled: 59 were approached, but 19 were absent, 2 had abnormal laboratory findings, and 2 declined consent (eFigure 1 in the Supplement). Absence occurred as a result of authorized leave or reassignment to another military unit. Patients were assigned generic study numbers without regard to case or control identity to mask the laboratory teams later assigned to analyze the specimens in Jakarta, Indonesia.

### Exposure Measurements

#### Primaquine

Quantitative exposure to primaquine in the clinical trial was estimated from plasma concentrations of primaquine obtained from population pharmacokinetic sampling regimens during that trial.^21^ The methods of high-performance liquid chromatography measurement and mathematical modeling of the area under the curve for primaquine are detailed in the eAppendix in the Supplement essentially as described elsewhere.^28,29,30^

#### *CYP2D6* Genotype

Patient DNA was isolated and purified from venous blood samples using a QIAamp DNA Mini Kit (Qiagen). After ascertainment of concentration (NanoDrop2000; Thermo Scientific) and purity (A260/A280) of the DNA samples, *CYP2D6* genotyping was performed using xTAG CYP2D6 kit, version 3 (Luminex).^18^ The kit probed sequences specific to *1, *2, *3, *4, *5, *6, *7, *8, *9, *10, *11, *15, *17, *29, *35, and *41 alleles. Manufacturer’s instructions were followed using a 9700 thermal cycler (PE Applied Biosystems) and the Luminex 200 xMAP instrument. xTAG Data Analysis Software analyzed the fluorometric readout and recorded *CYP2D6* allelic identity. *CYP2D6* genotype identified levels of enzymatic activity qualitatively by the genotyping kit manufacturer’s guidance (poor, intermediate, extensive, or ultra) or by the activity score model described by Gaedigk et al,^22^ with alleles scored as 0.0, 0.5, or 1.0. The sum of the 2 alleles gave activity scores of 0.0, 0.5, 1.0, 1.5, or 2.0 as individual predicted activity score phenotypes.

#### CYP2D6 Dextromethorphan Metabolizer Phenotype

Enrollment in this study immediately commenced after the conclusion of the clinical trial of primaquine,^21^ with 6 cohorts of approximately 10 patients being admitted to a temporary 10-bed research ward within the health clinic that served the army base. Admission was followed by a supervised, 24-hour, water-only fast as recommended.^24^ Supervised administration of 2 tablets each that contained 15 mg of dextromethorphan hydrobromide (Kimia Farma) then occurred, and patients were kept in the ward for another 24 hours with normal meals and liquids.^23,24,25^ Urine output from each patient during that period was collected as a pooled sample. Discharge from the ward at that time marked the end of participation. Urine samples were transferred to Jakarta, stored at −80°C, and analyzed by liquid chromatography for dextromethorphan and dextrorphan essentially as described elsewhere,^31,32^ with details provided in the eAppendix in the Supplement.

### Main Outcomes Analysis

In the instance of both *CYP2D6* genotype–determined activity score and the measured dextromethorphan-dextrorphan metabolic ratio, we aimed to estimate risk of relapse at given thresholds of CYP2D6 activity separating impaired from normal drug metabolism. Those thresholds were set as poor or intermediate vs extensive or ultrametabolizers (genotype-determined qualitative phenotype), a genotype-determined metabolizer activity score phenotype less than 1.5, and a log metabolic ratio of dextromethorphan-dextrorphan greater than −1.0. Genotype-determined activity scores of 0.0, 0.5, and 1.0 derive from genotypes almost universally classified qualitatively as poor or intermediate metabolizers, and those of 1.5 or greater are classified as extensive or ultrametabolizers.^22^ A population survey of 660 white and African American patients found 66 patients with urinary log dextromethorphan-dextrorphan metabolic ratios of −1.0 or higher (ie, the lowest 10% of expressed CYP2D6 metabolic activity values).^22^ We thus considered the applied thresholds of impaired vs normal CYP2D6 activity as objectively valid.

Three outcomes were assessed: (1) odds ratios (ORs) for relapse among patients classified as poor or intermediate metabolizers according to the *CYP2D6* genotyping kit compared with those classified as extensive or ultrametabolizers; (2) OR for relapse among patients with a genotype-determined activity score of 1.0 or less compared with those with higher scores; and (3) OR for relapse among patients with a log metabolic ratio of dextromethorphan to dextrorphan of greater than −1.0 compared with those with greater dextrorphan metabolism.

### Statistical Analysis

Unadjusted ORs (95% CIs) were calculated with the Fisher exact test to determine the association among the CYP2D6 phenotype, *CYP2D6* genotype, and log_10_ metabolic ratio of dextromethorphan to dextrorphan between patients who did and did not experience relapse. The Mann-Whitney *U* test was performed to compare the differences in mean genotype-determined activity scores between patients who did and did not experience relapse. Statistical analysis was performed using Stata software, version 12 (StataCorp). Two-sided *P* < .05 was considered to be significant in all analyses.

## Results

### Study Populations

The study included 21 cases (mean [SD] age, 30.5 [6.3] years; all male) and 36 controls (mean [SD] age, 29.0 [3.6] years; all male). Table 1 summarizes the essential demographic, clinical, and laboratory characteristics of the study participants. With regard to race/ethnicity, body weight, parasite counts at enrollment in the clinical trial, and blood schizontocidal treatment assignment, no statistically significant differences emerged. Patients who weighed less than 70 kg received a daily dose of 30 mg of primaquine, whereas those above that threshold received 45 mg; the number of controls who received the higher dose (20 of 36) was significantly higher than the number of cases (5 of 21) (*P* = .03). Nonetheless, no significant differences in primaquine population pharmacokinetic area under the curve values occurred between controls and cases (32.2 vs 32.6 μg · h/mL, *P* = .29) (Table 1). Primaquine exposure among cases and controls appeared to be equal and not dissimilar from that of the clinical trial population as a whole (eAppendix, eTable 1, and eTable 2 in the Supplement). Primaquine treatment failures occurred among all 3 randomized blood schizontocidal regimens, and all were represented equally among cases and controls (Table 1).

**Table 1.** Baseline Characteristics of Case Patients and Control Individuals^a^

Characteristic	Cases (Relapse)	Controls (No Relapse)	*P* Value
Race/ethnicity, No.			
Javanese	21	32	.29
Non-Javanese	0	4	
Age, mean (SD) [range], y	30.5 (6.3) [24-46]	29.0 (3.6) [24-46]	.81
Body weight, mean (SD), kg	69 (6.8)	70 (8.2)	.56
Parasite count, median (range), /μL	784 (16-7664)	688 (16-10 608)	.33
Blood schizontocide, No.			
Artesunate alone	8	13	.64
Artesunate-pyronaridine	8	11	
Dihydroartemisinin-piperaquine	5	12	
Primaquine administration			
30 mg/d (total dose, 420 mg)	16	16	.03
45 mg/d (total dose, 630 mg)	5	20	
Plasma primaquine population kinetics, median (range), μg · h/mL	32.6 (15-55)	32.2 (19-63)	.29

^a^ Baseline data at enrollment for the clinical trial are presented.^21^ All patients were male.

No correlation appeared between weight of cases and week of relapse (*r* = 0.1644; *P* = .23). An analysis of relapse risk stratified by weight and exposure to a total dose of primaquine phosphate of 420 mg (body weight <70 kg) vs 630 mg (body weight ≥70 kg) found no significant difference in frequency of relapse (15 of 32 vs 6 of 25; OR, 2.8; 95% CI, 0.88-8.83; *P* = .08). There was a higher frequency of impaired metabolizers among patients who weighed less than 70 kg (83 of 94 [88%]) compared with individuals who weighed more (48 of 86 [56%]) (*P* = .01).

### Odds of Relapse With Exposure to Impaired *CYP2D6* Genotype

Table 2 summarizes the 12 distinct *CYP2D6* genotypes found among the 57 patients evaluated. The OR of relapse with a classification of poor or intermediate CYP2D6 metabolizer phenotype derived was 7.5 (95% CI, 1.8-36; *P* = .002) compared with a classification of extensive metabolizer (Table 3). Six *CYP2D6* alleles (*1, *2, *4, *5, *10, and *41) occurred as 12 distinct genotypes, with model activity scores ranging from 0.0 to 2.0. Among 32 patients with genotypic activity scores of 1.0 or less, 18 had experienced relapse, whereas among the 25 with scores higher than 1.0, 3 had experienced relapse. The OR for relapse with an activity score of 1.0 or less was 9.4 (95% CI, 2.1-57.0; *P* = .001) compared with a score of 1.5 or higher (Table 3 and eAppendix and eFigure 2 in the Supplement). The timing of postprimaquine relapse did not correlate with genotype-determined activity scores (eAppendix and eFigure 3A in the Supplement).

**Table 2.** Cytochrome P450 2D6 Genetic Profiles and Predicted Phenotypes of Case Patients and Control Individuals Who Experienced Relapse

Genotype	Predicted Metabolizer Phenotype^a^	Predicted Activity Score^b^	Predicted Primaquine Metabolism Phenotype^c^	No. of Cases (Relapse)	No. of Controls (No Relapse)
*4/*5	Poor	0	Null	1	0
*5/*10	Intermediate	0.5	Impaired	5	3
*10/*10	Intermediate	1.0	Impaired	9	8
*10/*41	Intermediate	1.0	Impaired	2	2
*2/*5	Extensive	1.0	Impaired	1	0
*2/*4	Extensive	1.0	Impaired	0	1
*1/*10	Extensive	1.5	Normal	1	9
*2/*10	Extensive	1.5	Normal	2	6
*1/*41	Extensive	1.5	Normal	0	1
*1/*1	Extensive	2.0	Normal	0	3
*1/*2	Extensive	2.0	Normal	0	2
*2/*2	Extensive	2.0	Normal	0	1

^a^Per genotyping kit manufacturer’s guidance.

^b^Per the study by Gaedigk et al,^22^ in which 0.0 indicates null metabolic activity; 0.5 or 1.0, impaired activity; and 1.5 or 2.0, normal activity.

^c^Per impaired activity score threshold of less than 1.5 applied in the current study.

**Table 3.** Unadjusted Odds Ratios for Relapse Associated With Cytochrome P450 2D6 Genotype or Dextramethorphan Metabolizer Phenotype

Assessment	No. of Cases (Relapse)	No. of Controls (No Relapse)	Odds Ratio (95% CI)	*P* Value
Poor or intermediate metabolizer genotype				
Yes	17	13	7.5 (1.8-36)	.002
No	4	23		
Genotype-determined activity score <1.5				
Yes	18	14	9.4 (2.1-57)	.001
No	3	22		
Log dextromethorphan-dextrorphan metabolic ratio phenotype >−1.0				
Yes	20	19	18 (2.2-148)	.007
No	1	17		

### Odds of Relapse With Exposure to Impaired CYP2D6 Phenotype

The Figure illustrates individual patient values derived from the 24-hour pooled urine sample for the log metabolic ratio of dextromethorphan to dextrorphan. An analysis of correlation between genotype-determined activity score and log metabolic ratio of urinary dextromethorphan to dextrorphan proved to be highly significant (Spearman correlation coefficient = −0.683; *P* < .001) (eAppendix and eFigure 4 in the Supplement). The Figure illustrates the occurrence of 20 of 21 cases above the log metabolic ratio of dextromethorphan to dextrorphan of −1.0, along with 19 controls. Conversely, 18 patients had values below that threshold and 17 were controls. When the log of the metabolic ratio of dextromethorphan-dextrorphan was −1.0 or less, only 1 of 18 patients experienced relapse, whereas above that threshold (consistent with low metabolic activity), 20 of 39 patients experienced relapse. Patients with dextromethorphan to dextrorphan ratios greater than −1.0 were 18 times more likely to experience relapse than were those with ratios of −1.0 or less (95% CI, 2.2-148.0; *P* = .007) (Table 3). No correlation was found between urinary log metabolic ratio of dextromethorphan to dextrorphan and day of relapse (eAppendix and eFigure 3B in the Supplement).

**Figure.**
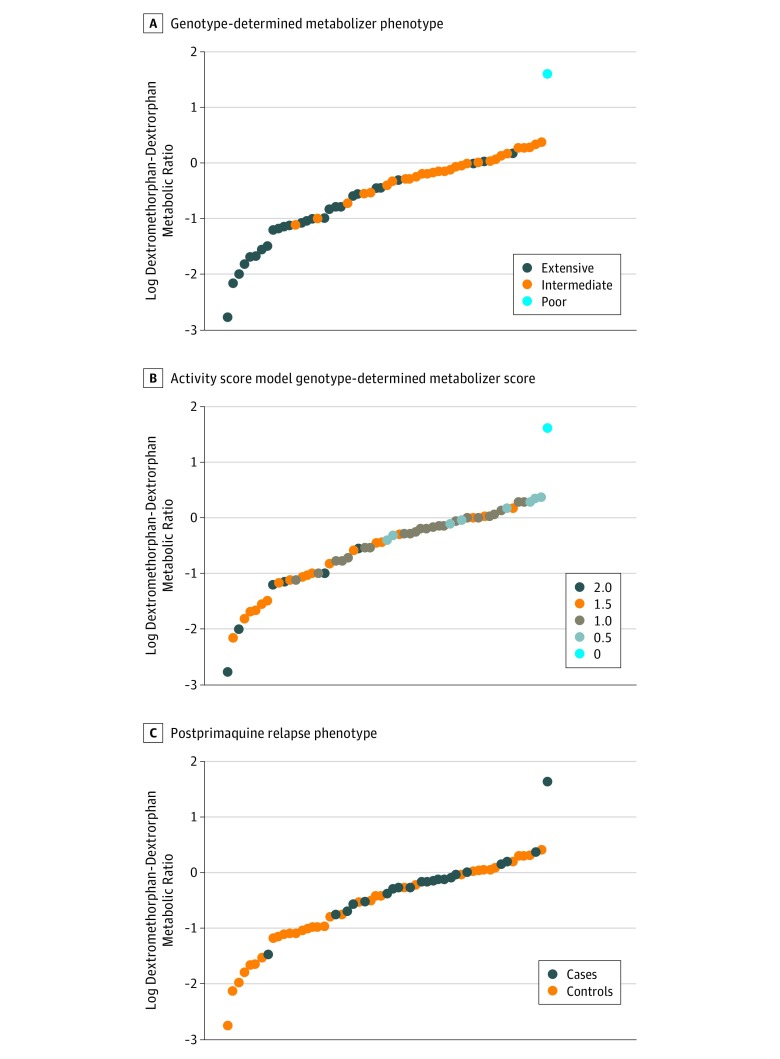
Estimates of Dextromethorphan Metabolism as the Log of Its Urinary Metabolic Ratio With Dextrorphan for All 57 Study Patients The estimates are arranged in ascending order (ie, left to right representing extensive, normal, impaired, and null). A, Distribution of genotype-determined qualitative cytochrome P450 2D6 (CYP2D6) metabolizer phenotypes as extensive, intermediate, or null. B, Distribution of genotype-determined quantitative activity scores of 2.0, 1.5, 1.0, 0.5, or 0 among patients. C, Distribution of controls and cases (no relapse vs relapse) along the continuum of log metabolic dextromethorphan-dextrorphan ratios.

## Discussion

Impaired CYP2D6 polymorphisms occurred in 20 of 21 Indonesian patients who experienced therapeutic failure of primaquine against relapse of *P vivax* malaria. These impaired polymorphisms were comparatively infrequent among patients with apparent therapeutic successes; 19 of 36 patients (OR of 18.0; 95% CI, 2.2-148.0; *P* = .007) had impaired CYP2D6 dextromethorphan-dextrorphan metabolizer phenotype. Impaired CYP2D6, whether identified by a genotype activity score less than 1.5 or a measured dextromethorphan-dextrorphan log metabolic ratio greater than −1.0, was associated with risk of primaquine therapeutic failure against relapse of *P vivax* malaria in this study, a finding that corroborates a large body of clinical and laboratory work posing and testing that hypothesis.^12^

These findings bear directly on treatment practice for patients with *P vivax* malaria. A single patient represented the null metabolizer genotype (*4/*5) and phenotype (log dextromethorphan to dextrorphan ratio >0) and experienced relapse. Any dose of primaquine in such patients may be futile against relapse, but among the 19 other cases classified as CYP2D6 impaired, higher or repeated doses would perhaps prove to be effective. Only 5 other patients had *4 or *5 alleles (4 of them did not experience relapse), but the sample of 36 controls included 28 (78%) who expressed the impaired *10 allele, as commonly appears in other Asian populations.^33^ This small sample of controls may or may not represent these allele frequencies among Javanese or other Indonesian ethnic groups, but the high frequency of *10 gives cause for concern regarding risk of therapeutic failure of primaquine against relapse in *P vivax* malaria caused by impaired metabolism. Daily doses of 0.5 mg/kg of primaquine in studies of primary chemoprophylaxis in travelers as long as 50 weeks were safe and well tolerated in nonfasted G6PD-normal and nonpregnant patients.^34^ Extending treatment beyond 14 days against relapse in patients with impaired (but not null) CYP2D6 activity may provide improved and sufficient efficacy with good safety and tolerability.

Routine screening of *CYP2D6* genotypes in patients with *P vivax* malaria is not currently practical because of the high cost and technical expertise required. Populations in areas of endemicity who carry high frequencies of impaired *CYP2D6* alleles would likely benefit from prolonged primaquine treatment as standard of care. However, such prolonged treatment could incur elevated risk of harm in undiagnosed G6PD-deficient patients and may also be impractical in terms of adequate adherence. In the broad context of *P vivax* malaria as it occurs in most people very often living in impoverished and isolated locations that lack laboratory services, the already substantial numbers of people who cannot receive safe and effective therapy against latent *P vivax* malaria thus further expands.^35^

These findings suggest that primaquine is a prodrug dependent on the CYP2D6 isozyme to generate a therapeutically active metabolite. When natural polymorphism of CYP2D6 resulted in null or impaired metabolism, risk of relapse increased substantially. This problem appeared to explain almost all therapeutic failures of primaquine against relapse of *P vivax* malaria acquired in eastern Indonesia by Indonesian patients. The single patient who experienced relapse despite seemingly normal *CYP2D6* genotype and CYP2D6 phenotype and adequate exposure to primaquine may represent rare evidence of apparently infrequent (<1% in the current trial) parasite resistance to primaquine. In patients able to adequately metabolize primaquine, this drug seems to retain high efficacy after 66 years of continuous clinical use as the sole therapeutic option against relapse of *P vivax* malaria.

### Limitations

Some controls may have lacked hypnozoites at the time of treatment with primaquine and would have been misclassified as treatment successes. Among patients in the clinical trials^20,21^ not treated with primaquine, 22% did not experience relapse. Inclusion of patients who lacked hypnozoites as controls would bias the OR for relapse associated with impaired CYP2D6 downward; thus, we considered the measured risk estimates to be conservative. The *CYP2D6* genotyping kit manufacturer’s instructions warn of erroneous default common allele calls with rare alleles, and the populations surveyed for design of those genetic probes included ethnic Japanese and Han Chinese as the only Asian people represented. We are not aware of any surveys of *CYP2D6* alleles among ethnic Javanese and thus cannot know the likelihood of rare allele misclassification by this method.

## Conclusions

More than 90% of the global burden of *P vivax* malaria occurs in South and Southeast Asia,^1^ regions of great human genetic and ethnic diversity. *CYP2D6* allele diversity and frequencies in these regions have not been adequately surveyed. The likely effects of potentially high frequencies of impaired *CYP2D6* metabolism alleles merit those assessments with regard to technical strategies for striving to eliminate *P vivax* malaria from the whole of the Asia-Pacific region. Preventing relapse in patients diagnosed with *P vivax* malaria is an essential element of the responsible clinical and public health management of this infection.^8^ Alternative chemotherapeutic or chemoprophylactic strategies for managing risk of relapse in patients unable to receive or benefit from primaquine therapy (G6PD-unknown patients, G6PD-deficient patients, pregnant women, lactating mothers, young infants, and CYP2D6-impaired patients) have not been conceived, optimized, or validated. Preventing relapse without 8-aminoquinoline treatment should be considered to be a neglected gap in malaria chemotherapeutics that is in urgent need of attention.

## Notes

Supplement. **eAppendix.** Supplementary Methods **eTable 1.** Primaquine Pharmacokinetics in Study Population vs. Clinical Trial Population **eTable 2.** Population Pharmacokinetic Areas Under the Curve (μg hr/mL) for Cases vs. Controls **eFigure 1.** Case and Control Recruitment **eFigure 2.** Case and Control Distribution Across CYP2D6 Activity Scores **eFigure 3.** CYP2D6 Genotype and Phenotype vs. Week of Relapse among Cases **eFigure 4.** Correlation of CYP2D6 Activity Scores and Dextramethorphan Metabolizer Phenotype **eReferences**
