# Supplementary material for: Association of Impaired Cytochrome P450 2D6 Activity Genotype and Phenotype With Therapeutic Efficacy of Primaquine Treatment for Latent Plasmodium vivax Malaria
Source: JAMA Netw Open. 2018 Aug 31;1(4):e181449. doi: 10.1001/jamanetworkopen.2018.1449 (PMC6324265; doi:10.1001/jamanetworkopen.2018.1449)
Supplement: Supplement. — eAppendix. Supplementary Methods eTable 1. Primaquine Pharmacokinetics in Study Population vs. Clinical Trial Population eTable 2. Population Pharmacokinetic Areas Under the Curve (μg hr/mL) for Cases vs. Controls eFigure 1. Case and Control Recruitment eFigure 2. Case and Control Distribution Across CYP2D6 Activity Scores eFigure 3. CYP2D6 Genotype and Phenotype vs. Week of Relapse among Cases eFigure 4. Correlation of CYP2D6 Activity Scores and Dextramethorphan Metabolizer Phenotype eReferences [file jamanetwopen-1-e181449-s001.pdf]

Supplementary Online Content

Baird JK, Louisa M, Noviyanti R, et al. Association of Impaired Cytochrome P450 2D6 Activity Genotype and Phenotype With Therapeutic Efficacy of Primaquine Treatment for Latent *Plasmodium vivax* Malaria. *JAMA Netw Open.* 2018;1(4):e181449. doi:10.1001/jamanetworkopen.2018.1449

eAppendix. Supplementary Methods

eTable 1. Primaquine Pharmacokinetics in Study Population vs. Clinical Trial Population

eTable 2. Population Pharmacokinetic Areas Under the Curve (µg hr/mL) for Cases vs. Controls

eFigure 1. Case and Control Recruitment

eFigure 2. Case and Control Distribution Across CYP2D6 Activity Scores

eFigure 3. CYP2D6 Genotype and Phenotype vs. Week of Relapse among Cases

eFigure 4. Correlation of CYP2D6 Activity Scores and Dextramethorphan Metabolizer Phenotype

eReferences

This supplementary material has been provided by the authors to give readers additional information about their work.

eAppendix. Supplementary Methods

Primaquine Plasma Concentration.

Primaquine in plasma were quantified using HPLC-Photodiode Array Detector (Alliance e2695 HPLC system, PDA 2998 detector, Waters, USA) according to the method of Ward [e1], with slight modification. Detection wavelength was set at 263 nm. Mobile phase used consisted of paired ion chromatography (PIC) B5 solution 10%: water (HPLC grade) 45%: methanol 20%: acetonitrile 25%. The separation of primaquine in plasma were done on Phenomenex Gemini C18 3 μm 4.6 x 100 mm analytical column and Phenomenex Gemini C18 4.6 x 30 mm guard column. Column temperature was set at 35^0C. To a 200-µL plasma sample, 5 μg/mL 8-/3-amino-1-methylpropylamino/-6-methoxyquinoline diphosphate (internal standard) was added and the extraction was done using ethylacetate:hexane (1:9). The supernatant was transferred to another tube and evaporated under a stream of nitrogen gas until dry. Then 150 µL of mobile phase were added to dissolve the residue and 30 µL were injected automatically onto the HPLC system for analysis.

Data were analyzed using nonlinear mixed effect modeling method. Sparse plasma sample was drawn at eight time slots relative to dosing (pre-dose, hour 0-2, h 2-6, h 6-24, day 9-10, day-11, day-14, day-22). Modeling and simulation were performed using NONMEM v.7.4.1 (ICON Development Solutions) on Windows 8.1 with G95 Fortran Compiler. The results were processed using Pirana v.2.9.4. and R statistics v.0.99.903 [e2]. We investigated possible compartment dispositions, absorption models, inter-individual variability implemented as exponential on all parameters, and used additive error on log transformed drug concentration as residual variability. Body weight was tested as an allometric function on clearance and volume distribution parameters, and concomitant antimalarial to build the covariate model. Objective function values were used to compare two hierarchical models (p = 0.05) [e3].

Urinary Dextramethorphan/Dextrorphan Ratio.

Dextramethorphan (DXM) and dextrorphan (DX) were quantified using HPLC-fluorescence detection (Alliance e2695 HPLC system, with 2475 FLR Detector, both from Waters, USA) according to the method of Lam [e4], with slight modification. Emission filter was 280nm and excitation wavelength was 305nm. Mobile phase was 30% acetonitrile and 70% 0.01M heptane sulfonic in 0.01M KH_2PO_4. The separation of DXM and DX in urine was on X-Bridge Phenyl analytical column (150 mm x 4.6mm) with a particle size of 5-µm (Waters, USA). Column temperature was 40^0C. Extraction of DXM and DX in urine was by the method of Daali [e5], with modification. To 500 µL urine, 75 µL of HCl 10% was added, followed by 50-µl aliquot of internal standard solution (codeine, 100 µg/mL). 2000 U of ß-glucuronidase was then added and vortexed for 15 seconds, and incubated at 37^0C for 18 hours. The hydrolyzed urine samples were then alkalinized with KOH 5N 100 µL and 500 µL Na_2CO_3 1M was added and vortexed, followed by the addition of 4 mL hexane:ethylacetate (1:1). This solution was vigorously mixed for 15 minutes and then centrifuged (3000rpm; 10min). The upper organic layer was discarded and the lower aqueous phase kept in the dark of 20 minutes to ensure complete elimination of organic solvents. Forty microliters were injected into the HPLC instrument by an automated system.

eTable 1. Primaquine Pharmacokinetics in Study Population vs. Clinical Trial Population

Primaquine pharmacokinetic characteristics among clinical trial population (n=174) compared to subjects in the current study (n=57: 36 controls and 21 cases).

Parameters	Population	Subset Subjects	p-value
Number of subjects	174	57	-
Clearance (liter per hour)	22.2	22.4	0.875
Volume distribution (liter)	216	228	0.940
Absorption rate constant (per hour)	0.584	0.620	0.449

© 2018 Baird JK et al. *JAMA Network Open.*

eTable 2. Population Pharmacokinetic Areas Under the Curve (μg h/mL) for Cases vs. Controls

Population pharmacokinetic area under the curve for 14 days primaquine daily dosing of 0.5mg/kg among clinical trial population (n=174; 26 relapsers and 148 non-relapsers) and the current study population (n=57; 21 relapsing cases and 36 non-relapsing controls).

Subject	Case (Relapse)	Control (No Relapse)	p-value
Population (N=174)	30.4 (mean)	32.5 (mean)	
	31.2 (median)	31.6 (median)	0.342
Subset Subject (N=57)	30.3 (mean)	34.3 (mean)	
	32.6 (median)	32.4 (median)	0.254

© 2018 Baird JK et al. *JAMA Network Open.*

eFigure 1. **Case and Control Recruitment**

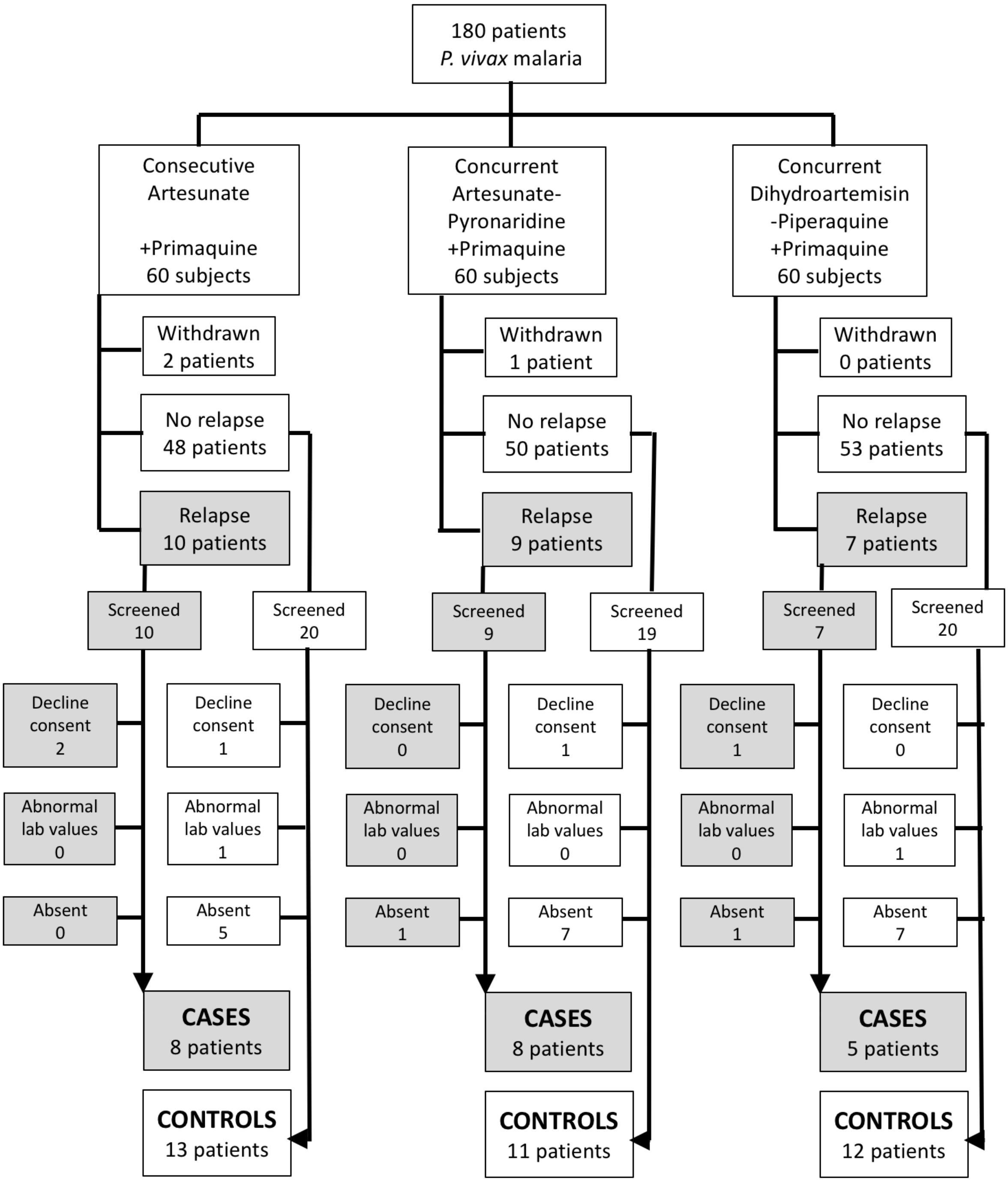

© 2018 Baird JK et al. *JAMA Network Open.*

eFigure 2. Case and Control Distribution Across CYP2D6 Activity Scores

Distribution of controls (black bars) and cases (red bars) along AS-Model activity scores. The median (mean; standard deviation) scores of 1.0 (0.905; 0.375) and 1.5 (1.348; 0.428) for cases and controls, respectively, differed significantly (P=0.0004).

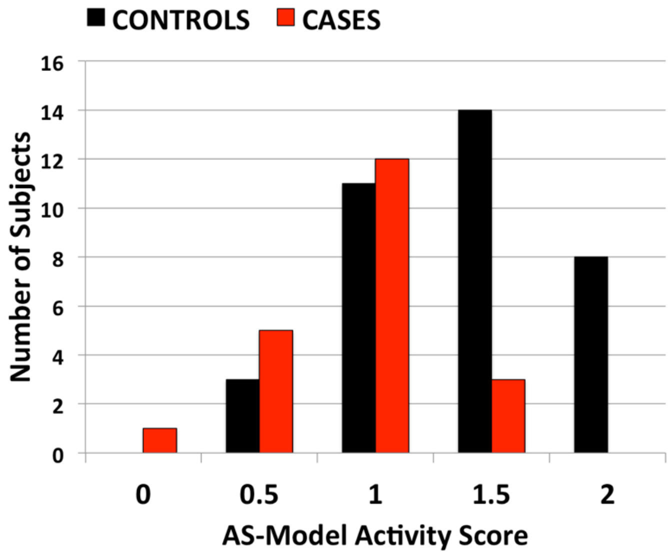

eFigure 3. CYP2D6 Genotype and Phenotype vs. Week of Relapse among Cases

Lack of correlation between genotype-predicted CYP2D6 activity score (panel A, left) or urinary log metabolic ratio of DXM/DX (panel B, right) and week of relapse event during the year following acute vivax malaria.

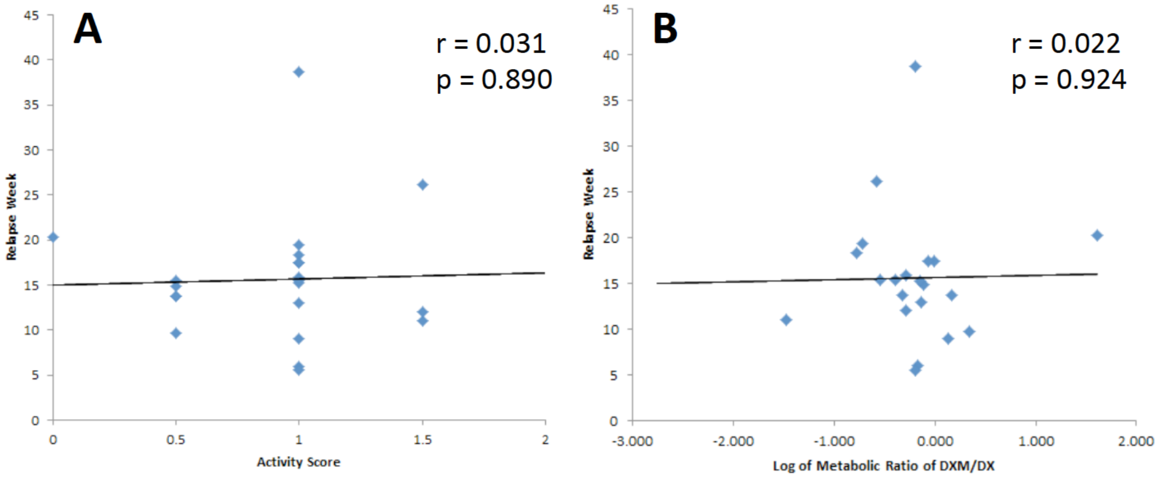

© 2018 Baird JK et al. *JAMA Network Open.*

eFigure 4. **Correlation of CYP2D6 Activity Scores and Dextramethorphan Metabolizer Phenotype**

Significant correlation between genotype-predicted activity score and log metabolic ratio of DXM/DX in 24hr pooled urine samples from all 57 subjects categorized according to qualitative genotype-predicted phenotype (extensive, blue; intermediate, orange; or poor, green) (panel A), or cases (black) and controls (white) (panel B).

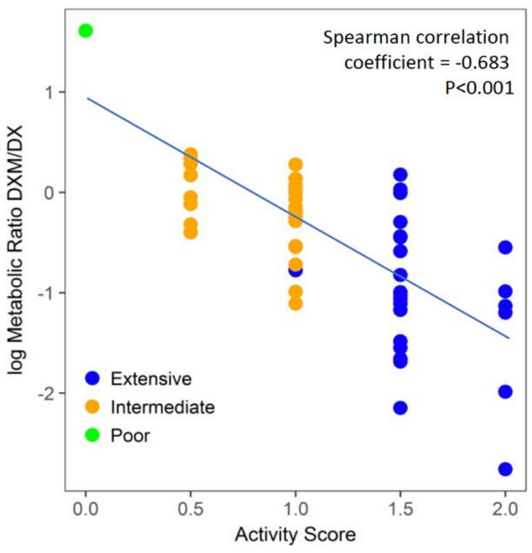

eReferences

S1. Ward SA, Edwards G, Orme MLE, Breckenridge AM. Determination of primaquine in biological fluids by reversed-phase high performance liquid chromatography. J Chromatography. 1984; 305:239 – 43.

S2. Mould DR, Upton RN. Basic concepts in population modeling, simulation, and model-based drug development – Part 2: introduction to pharmacokinetic modeling methods. Pharmacometrics & Systems Pharmacology 2013; 2: e38.

S3. Keizer RJ, Karlsson MO, Hooker A. Modeling and simulation workbench for NONMEM: tutorial on Pirana, PsN, and Xpose. Pharmacometrics & Systems Pharmacology 2013; 2: e50.

S4. Lam YW, Rodriquez SY. High performance liquid chromatography determination of dextromethorphan and dextrorphan for oxidation phenotyping by fluorescence and ultraviolet detection. Ther Drug Monit. 1993; 15(4):300-4.

S5. Daali Y, Cherkaoui S, Doffey-Lazeyras F, Dayer P, Desmeules JA. Development and validation of a chemical hydrolysis method for dextromethorphan and dextrorphan determination in urine samples: application to the assessment of CYP2D6 activity in fibromyalgia patients. J Chromatogr B Analyt Technol Biomed Life Sci. 2008; 861(1):56 – 63.

© 2018 Baird JK et al. *JAMA Network Open.*
